# Prescription Trends of Initial Pharmacotherapy for Benign Prostatic Hyperplasia Among Treatment‐Naïve Patients in South Korea: A Retrospective Analysis

**DOI:** 10.1111/luts.70030

**Published:** 2025-09-05

**Authors:** Yeon Hee Kim, Nam Kyung Je

**Affiliations:** ^1^ College of Pharmacy Pusan National University Busan Republic of Korea; ^2^ Research Institute for Drug Development Pusan National University Busan Republic of Korea

**Keywords:** 5‐alpha‐reductase inhibitors, alpha‐1 blockers, benign prostate hyperplasia, initial pharmacotherapy, prescription trends

## Abstract

**Background:**

Benign prostatic hyperplasia (BPH) is a common urological condition in aging men that causes lower urinary tract symptoms. Pharmacotherapy is central to BPH management; however, considering updated guidelines, recent prescription trends remain insufficiently explored. This study aimed to assess initial pharmacotherapy trends in patients newly diagnosed with BPH.

**Methods:**

This cross‐sectional analysis used 2015–2020 data from the Health Insurance Review and Assessment Service to examine the trends and influencing factors of BPH drug utilization among South Korean patients aged ≥ 40 years with no prior history of BPH. Subgroup analyses were performed by categorizing patients into five age groups (40–49, 50–59, 60–69, 70–79, and ≥ 80 years) to evaluate age‐specific prescription patterns.

**Results:**

Of the 1,445,144 patients newly diagnosed with BPH, 1,336,695 (92.4%) initiated treatment within 60 days of diagnosis. Among those treated, 54.9% received α‐blocker (AB) monotherapy and 17.9% received 5α‐reductase inhibitor (5‐ARI)/AB combinations. Use of 5‐ARI/AB combinations increased with age, from 8.0% in patients in their 40s to 25.4% in those aged ≥ 80 years. Tamsulosin (a selective AB), dutasteride (a 5‐ARI), and mirabegron (a β3‐agonist) were the most frequently prescribed agents.

**Conclusion:**

Recent American Urological Association (AUA) guidelines recommend combination therapy as an effective strategy for reducing the risk of BPH‐related complications. Although the largest proportion of patients was prescribed AB monotherapy, the growing adoption of combination therapy in South Korea, particularly among older age groups, suggests a shift toward more guideline‐concordant and effective therapeutic approaches.

## 1 Introduction

Benign prostatic hyperplasia (BPH) is a common condition in aging men; it is characterized by noncancerous enlargement of the prostate gland and frequently leads to lower urinary tract symptoms (LUTS), which significantly impair quality of life [1, 2]. Globally, the prevalence and treatment demand of BPH continue to rise, generating substantial healthcare and economic burdens [3].

In general, BPH treatment includes lifestyle modifications, behavioral interventions, pharmacotherapy, and surgery [4]. For patients exhibiting mild symptoms, watchful monitoring without immediate active intervention is typically recommended [5, 6]. However, pharmacotherapies or surgery should be considered for those experiencing moderate to severe LUTS [5, 7]. Over the past decades, the advent of α‐blockers (AB) and 5α‐reductase inhibitors (5‐ARI) has reduced the need for surgery in many patients with BPH, making pharmacotherapy the first‐line treatment [8, 9].

American Urological Association (AUA) guidelines have progressively expanded pharmacological treatment options for BPH/LUTS. Since 2003, they have recommended AB, 5‐ARI, and their combination [10]. The 2010 update added anticholinergics for storage symptoms [4], and the 2023 revision has further specified indications for 5‐ARI/AB combination therapies in cases of significant prostatic enlargement. Additionally, anticholinergics or β3‐agonists in combination with AB have been conditionally recommended for patients predominantly experiencing storage symptoms, and low‐dose tadalafil combined with AB or 5‐ARI has been introduced as another therapeutic option, irrespective of erectile dysfunction status [5]. The 2016 Korean guidelines closely align with these recommendations, advocating for 5‐ARI/AB combination therapies for patients with enlarged prostates and the cautious use of anticholinergics for patients predominantly experiencing storage symptoms; however, the use of β3‐agonists is not explicitly addressed [6].

Given these evolving guidelines and the introduction of novel pharmacological agents, evaluating current prescription trends in BPH treatment is essential. Although previous studies have assessed general trends in BPH medication use, detailed analyses reflecting current AUA guidelines and identifying differences across age groups remain limited. Understanding these trends is critical for aligning real‐world clinical practices with contemporary guidelines and for optimizing patient outcomes.

Therefore, this study aimed to analyze the prescription patterns and temporal trends of initial pharmacotherapy regimens among patients newly diagnosed with BPH. Specifically, we examined how recent guidelines influenced clinical practice and assessed variations in prescription trends across different age groups, thereby providing valuable insights for future clinical decision‐making and guideline development.

## 2 Methods

### 2.1 Data Source

This cross‐sectional study obtained data from the Health Insurance Review and Assessment Service (HIRA) database; this dataset included detailed information on medical claims, healthcare services, and prescriptions covered by the National Health Insurance (NHI) System from all healthcare providers. The dataset was obtained from HIRA as part of a customized research project (No. M20240825001).

Diagnostic codes were sourced from the 6th to 8th editions of the Korean Classification of Diseases (KCD‐6, KCD‐7, and KCD‐8) (Table S1). Drug codes were obtained from the HIRA Reimbursement Drug List (Table S2).

### 2.2 Study Population

**FIGURE 1:**
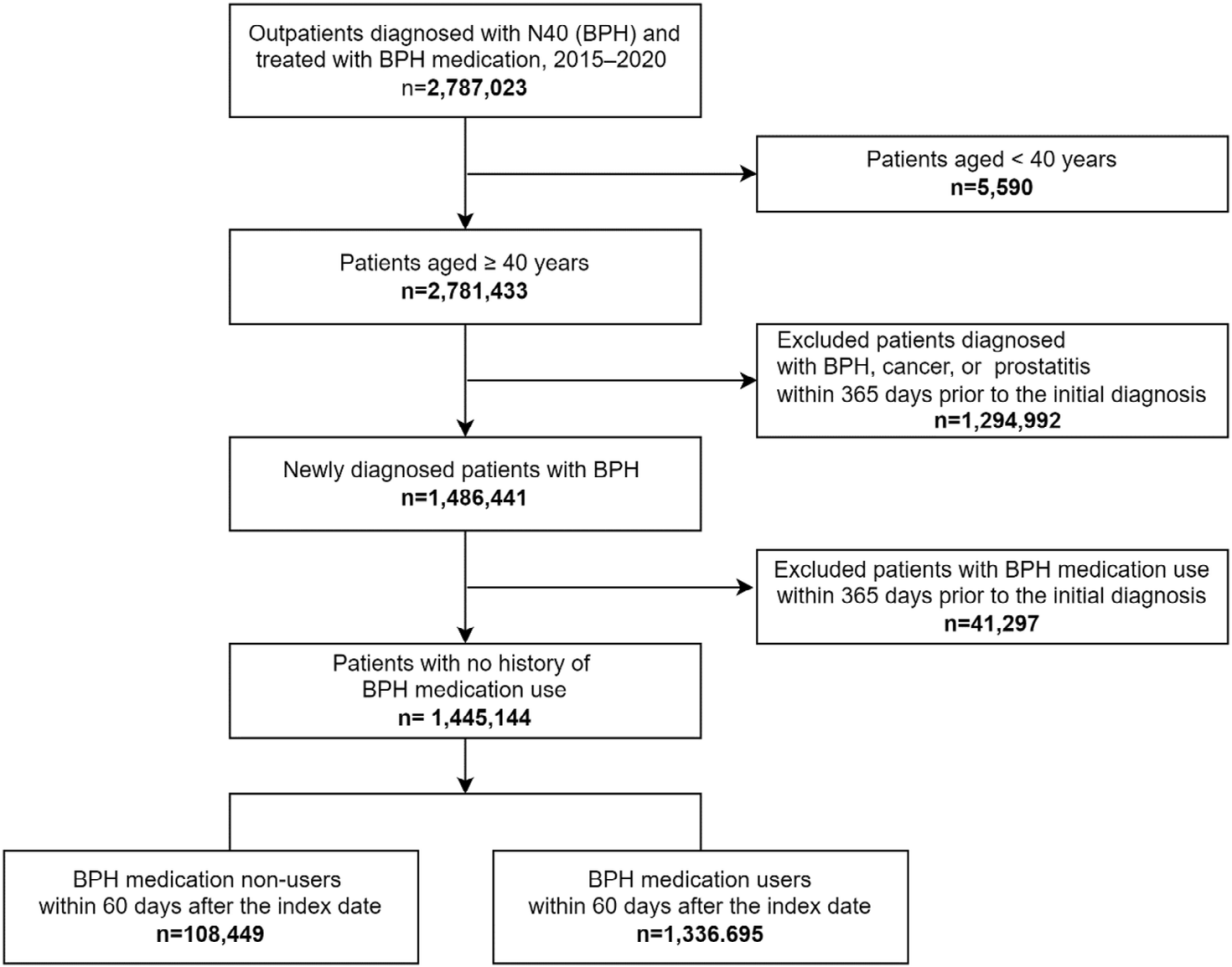
Study population flow chart. BPH, benign prostatic hyperplasia.

This cohort of treatment‐naïve patients with BPH included patients aged ≥ 40 years who were newly diagnosed with BPH (KCD‐6 to KCD‐8: N40) between January 2015 and December 2020 (Figure 1). The index date was defined as the date of first diagnosis. Patients were excluded if they had a prior diagnosis of BPH, cancer, or prostatitis, or had a history of BPH medication use during the 365 days before the index date. To analyze the first treatment selection, only patients who initiated BPH treatment within 60 days of diagnosis were included.

Comorbidities from the year before the index date were collected, including hypertension, atrial fibrillation, heart failure, ischemic heart disease including myocardial infarction, diabetes, peripheral vascular disease, dyslipidemia, transient ischemic attack, renal failure, angina, cerebrovascular disease including stroke, gout/hyperuricemia, and osteoporosis.

The presence of BPH‐related complications was classified based on the detailed KCD codes. N400 was categorized as “hyperplasia of the prostate without complications”, whereas N401, N402, N403, and N408 were classified as “hyperplasia of the prostate with complications”.

In this study, insurance status was categorized as NHI or Medical Aid (MedAid). The NHI covers most of the population, whereas MedAid is a government‐supported program for low‐income individuals. Patients covered by Patriots and Veterans Insurance were included in the MedAid category.

The study population was categorized based on several key factors. Age was grouped into five categories: 40–49, 50–59, 60–69, 70–79, and ≥ 80 years. Healthcare facility type, defined by the level of the prescribing institution, was classified as tertiary hospitals, general hospitals, hospitals, or clinics. The geographical region of the prescribing facility was categorized as capital, metropolitan, or other regions. Lastly, prescriber specialties were classified as internal/family medicine, dermatology, urology, or others.

### 2.3 Drugs for BPH/LUTS

BPH/LUTS medications were categorized into four classes: AB, including tamsulosin, alfuzosin, silodosin, naftopidil, terazosin, and doxazosin; 5‐ARI, including finasteride and dutasteride; phosphodiesterase type 5 inhibitors (PDE5I), specifically tadalafil; and overactive bladder agents (OAB), including anticholinergic agents, such as solifenacin, propiverine, tolterodine, flavoxate, fesoterodine, imidafenacin, oxybutynin, and trospium, as well as β3‐agonists, such as mirabegron.

Treatment regimens were categorized into seven groups: AB, 5‐ARI, and OAB monotherapy; and combination therapies, including AB/OAB, 5‐ARI/AB, 5‐ARI/OAB, and 5‐ARI/AB/OAB (triple therapy). The use of PDE5I was not analyzed because it was not covered by insurance in South Korea during the study period.

### 2.4 Statistical Analysis

Descriptive statistics (counts and percentages) were used to summarize baseline characteristics. To examine the first‐line pharmacotherapy choices in treatment‐naïve patients newly diagnosed with BPH, the Cochran–Armitage test was used to assess linear time trends in drug therapy during the study period. Subgroup analyses were conducted based on age group.

All analyses were performed using R software (version 3.5.1; R Foundation for Statistical Computing, Vienna, Austria). A *p*‐value < 0.05 was considered statistically significant.

## 3 Results

### 3.1 Baseline Characteristics

A total of 1,336,695 patients newly diagnosed with BPH, who initiated drug treatment within 60 days of diagnosis, were analyzed. The median age of the patients was 61 years (interquartile range: 54–69 years). The most represented age group was 60–69 years, accounting for 31.8% of the total, followed by 50–59 (30.6%), 70–79 (18.5%), 40–49 (13.2%), and ≥ 80 years (5.9%).

Regarding BPH‐related complications, 74.8% of the patients were categorized as BPH without complications, whereas 25.2% had complications. Hypertension (40.0%), dyslipidemia (40.0%), diabetes (23.4%), and peripheral vascular disease (12.4%) were the most frequently observed comorbidities.

In terms of insurance status, 95.4% were covered by NHI, whereas 4.6% were enrolled in MedAid. Most prescriptions (86.7%) were issued by urologists, and clinics were the predominant healthcare setting (74.8%). Geographically, 27.8% of the prescribing facilities were located in metropolitan areas, 19.4% in the capital region, and 52.8% in non‐metropolitan and non‐capital areas (Table 1).

**TABLE 1:** Baseline characteristics of the study population.

Variables	*N* (%)
Overall	1 336 695
Age (years, median [1QR; 3QR])	61.0 [54.0; 69.0]
Age group (years)	
40–49	176 495 (13.2)
50–59	408 985 (30.6)
60–69	425 712 (31.8)
70–79	246 962 (18.5)
≥ 80	78 541 (5.9)
Complication status	
Uncomplicated	1 000 563 (74.8)
Complicated	336 132 (25.2)
Comorbidity	
Hypertension	534 722 (40.0)
Dyslipidemia	535 190 (40.0)
Diabetes mellitus	312 967 (23.4)
Peripheral vascular disease	165 569 (12.4)
Angina pectoris	117 462 (8.8)
Gout/hyperuricemia	85 048 (6.4)
Cerebrovascular disease	60 063 (4.5)
Ischemic heart disease	55 210 (4.1)
Heart failure	39 879 (3.0)
Osteoporosis	38 296 (2.9)
Renal failure	33 063 (2.5)
Atrial fibrillation	30 256 (2.3)
Transient ischemic attack	16 506 (1.2)
Insurance	
NHI	1 275 818 (95.4)
MedAid/PVI	60 877 (4.6)
Prescriber specialty	
Internal/family medicine	93 704 (7.0)
Dermatology	35 551 (2.6)
Urology	1 158 432 (86.7)
Others	49 008 (3.7)
Prescribing facility type	
Tertiary hospital	61 884 (4.6)
General hospital	237 335 (17.8)
Hospital	38 167 (2.8)
Clinic	999 309 (74.8)
Region of prescribing facility	
Capital	259 842 (19.4)
Metropolitan	371 680 (27.8)
Other	705 173 (52.8)
Year	
2015	263 705 (19.7)
2016	251 067 (18.8)
2017	224 487 (16.8)
2018	218 355 (16.3)
2019	204 634 (15.3)
2020	174 447 (13.1)

Abbreviations: MedAid, Medical Aid; NHI, National Health Insurance; PVI, Patriots and Veterans Insurance; QR, Quartile Range.

### 3.2 Overall Trends in Initial BPH/LUTS Drug Treatment Regimens

During the study period, AB monotherapy (54.9%) was the most commonly prescribed regimen, followed by dual therapy with 5‐ARI/AB (17.9%) and AB/OAB (14.4%), 5‐ARI monotherapy (8.1%), 5‐ARI/AB/OAB (2.6%) triple therapy, OAB monotherapy (1.8%), and 5‐ARI/OAB (0.3%) dual therapy.

#### 3.2.1 AB Monotherapy

From 2015 to 2020, the use of tamsulosin gradually increased from 63.3% to 73.0%, whereas the use of terazosin and doxazosin declined from 9.8% to 5.7% and from 4.4% to 1.8%, respectively. Alfuzosin, naftopidil, and silodosin also showed a gradual decline in use over time (*p* < 0.001), as illustrated in Figure S1a.

#### 3.2.2 5‐ARI/AB Dual Therapy

Although dual therapy with 5‐ARI/AB was the second most commonly prescribed regimen, its overall utilization gradually decreased from 19.3% in 2015 to 13.7% in 2020 (*p* < 0.001). However, among the 5‐ARI/AB regimens, the use of dutasteride/tamsulosin significantly increased from 10.2% to 27.7%. In contrast, the use of finasteride/tamsulosin and finasteride/alfuzosin declined from 53.7% to 47.0% and from 10.0% to 5.6%, respectively (Figure S1b).

#### 3.2.3 AB/OAB Dual Therapy

The prescription of AB/OAB regimens exhibited significant growth, from 10.1% in 2015 to 20.6% in 2020 (*p* < 0.001). Among the combinations within this category, tamsulosin/mirabegron prescriptions showed the greatest increase, rising from 8.9% to 51.7%. Other combinations such as naftopidil/mirabegron and alfuzosin/mirabegron also demonstrated upward trends. However, the use of anticholinergics in AB/OAB regimens decreased over the study period (Figure S1c).

#### 3.2.4 5‐ARI Monotherapy

Overall, the independent use of 5‐ARI gradually declined from 8.4% in 2015 to 5.1% in 2020 (*p* < 0.001). Finasteride remained the predominant 5‐ARI, although its use decreased from 86.1% to 65.6%, whereas dutasteride use increased from 13.9% to 34.4% (Figure S1d).

#### 3.2.5 5‐ARI/AB/OAB Triple Therapy

Although it comprised a smaller proportion of the total prescriptions (2.6%), triple therapy use showed a consistent upward trend (*p* < 0.001). The combination of finasteride/tamsulosin/mirabegron exhibited a notable growth in use, from 5.2% in 2015 to 25.8% in 2020. Other triple combinations such as dutasteride/tamsulosin/mirabegron and finasteride/tamsulosin/solifenacin showed similar upward trends (Figure S1e).

#### 3.2.6 OAB Monotherapy

OAB prescriptions remained relatively stable, constituting 1.4% of prescriptions in 2015 and 2.2% in 2020 (*p* < 0.001). Mirabegron emerged as the most frequently used OAB, increasing from 11.3% to 64.9%. The use of most other OAB medications decreased significantly (Figure S1f).

#### 3.2.7 5‐ARI/OAB Dual Therapy

5‐ARI/OAB regimens were rarely used; however, prescriptions, including mirabegron, showed a slight upward trend during the study period (*p* < 0.001) (Figure S1g).

### 3.3 Age‐Specific Trends in Initial Drug Regimen

Given the clinical significance of patient age, we analyzed prescription trends by age group. AB monotherapy remained predominant across all age groups but showed declining trends; for example, use declined from 58.6% to 53.0% in patients in their 40s and from 61.2% to 56.0% in those in their 50s.

5‐ARI/AB dual therapy was more common in older patients, with the highest use in patients aged ≥ 80 (25.4%) and 70–79 (24.0%) years; decreasing trends were observed in all age groups.

AB/OAB dual therapy increased across all age groups, with the greatest increase in younger patients, from 10.9% to 24.1% and from 10.3% to 21.1% in patients in their 40s and 50s, respectively.

5‐ARI monotherapy use decreased with advancing age; its use was highest in patients aged 40–49 years (17.8%) and lowest in those aged ≥ 80 years (3.4%), declining significantly over the study period.

Triple therapy (5‐ARI/AB/OAB) use showed slight increases, particularly among older patients (70–79 years: 3.5%, ≥ 80 years: 4.0%).

**FIGURE 2:**
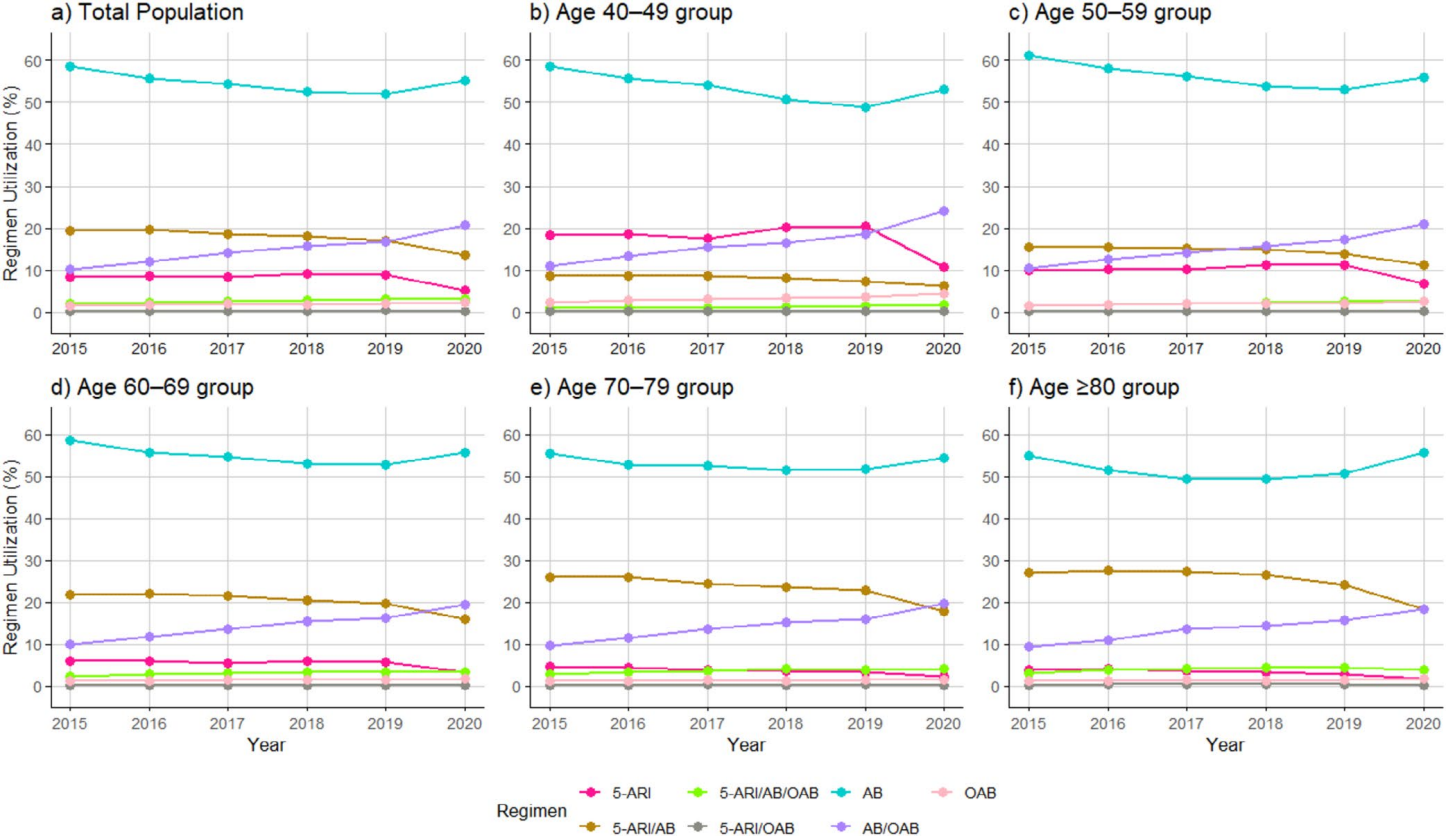
Trends in BPH regimen use by age group. AB, α‐blockers; BPH, benign prostatic hyperplasia; OAB, overactive bladder agents; 5‐ARI, 5α‐reductase inhibitors.

OAB monotherapy and 5‐ARI/OAB were rarely prescribed across all age groups, with slight increases in OAB monotherapy use among younger patients (40–59 years) (Figure 2).

## 4 Discussion

This study analyzed the prescription trends of the initial pharmacotherapy regimens of patients newly diagnosed with BPH between 2015 and 2020. Consistent with previous research, AB monotherapy remained the most frequently prescribed initial regimen, although its utilization gradually declined over the study period [11, 12]. Within this category, increases in tamsulosin prescriptions aligned with earlier findings of a marked rise following its FDA approval in 1997; this reflects clinicians' preference for newer selective AB, likely due to fewer side effects, particularly orthostatic hypotension, compared with non‐selective agents [12, 13]. Interestingly, while tamsulosin remains the predominant AB in South Korea, silodosin has become the most frequently prescribed agent in Japan [14]. This difference may stem from variations in clinical priorities; for example, Japanese urologists may place greater emphasis on symptom control, particularly for storage symptoms, as silodosin provides superior relief of such symptoms due to its high α1_A_ receptor selectivity [15]. Conversely, concerns about sexual side effects, especially ejaculatory dysfunction, may have limited the use of silodosin in South Korea and other countries; instead, tamsulosin is generally favored for its more balanced efficacy and tolerability profile [16]. These cross‐national differences reflect the influence of diverse factors, including regulatory approval timing, drug pricing, national clinical guidelines, patient preferences, and healthcare system characteristics. Understanding these variations can help contextualize local prescription trends and inform personalized pharmacotherapeutic strategies.

In contrast with AB monotherapy, the use of 5‐ARI monotherapy remained relatively low and decreased slightly between 2015 and 2020. Historical trends indicate that 5‐ARI has been steadily prescribed since its introduction, stabilizing after an initial increase in the early 2000s [11, 12, 13]. However, a growing shift from finasteride to dutasteride has been observed since the FDA approval of dutasteride in 2001. This shift is likely driven by differences in clinical outcomes; multiple real‐world studies have reported that dutasteride use is associated with a lower risk of BPH‐related complications, particularly acute urinary retention (AUR) and prostate‐related surgery. In an American managed care population, patients receiving dutasteride were less likely to experience AUR and require surgery than those treated with finasteride [17]. Similarly, a Dutch cohort study found that dutasteride users had a significantly lower risk of BPH‐related prostate surgery than finasteride users (hazard ratio: 0.75, 95% confidence interval: 0.56–0.99), particularly among men managed by urologists [18]. The consistency across different populations suggests that clinical superiority in reducing the risk of AUR and surgical intervention may explain the increasing global preference for dutasteride, regardless of the modest prescription rates of 5‐ARIs for BPH treatment.

A key observation from our analysis was the overall increase in the use of combination therapy compared with monotherapy for the management of BPH. This reflects the growing recognition of the multifactorial nature of LUTS and the need for more comprehensive symptom control. Within this broader trend, we observed notable shifts in the prescribed regimens. Specifically, the use of 5‐ARI/AB combination therapy decreased during the study period, despite robust evidence from landmark trials such as MTOPS and CombAT, which demonstrated superior efficacy in patients with enlarged prostates (≥ 30 g prostate volume) [19, 20]. Nonetheless, the substantial increase in dutasteride/tamsulosin prescriptions suggests that clinicians may still favor selective combination regimens offering complementary pharmacological effects and improved tolerability.

In contrast with 5‐ARI/AB dual therapy, we found a significant increase in AB/OAB combinations, particularly those involving the β3‐agonist mirabegron. This upward trend may reflect the growing clinical confidence in mirabegron's superior safety and efficacy profile compared with anticholinergics, particularly in elderly patients [5]. A meta‐analysis comparing AB/OAB regimen with AB monotherapy supports this observation, demonstrating that early combination therapy significantly improves storage symptoms, urgency, and quality of life [21]. Furthermore, a randomized trial found that the tamsulosin/mirabegron regimen offered superior symptom relief over tamsulosin alone [22]. Concurrently, the use of anticholinergic agents steadily declined, likely due to increased awareness of their cognitive and systemic side effects in older adults [23].

Age‐stratified analyses revealed meaningful differences in BPH regimen utilization, reflecting the evolution of clinical priorities and symptom profiles with age. Older patients (≥ 60 years) were consistently more likely to be prescribed combination therapy, particularly 5‐ARI/AB and AB/OAB regimens. This is likely associated with the greater severity of symptoms, prostate enlargement, and risk of progression in this group, in which monotherapy often fails to achieve adequate control [20, 24]. The preference for combination regimens aligns with the evidence that dual‐target therapy provides synergistic benefits in managing both voiding and storage symptoms in older populations [25].

Interestingly, the data showed a gradual increase in the use of AB/OAB combinations among the younger cohorts (40s and 50s). Although traditionally considered less symptomatic, these groups may increasingly report aggravating storage symptoms that are often underrecognized. This upward trend may reflect shifting diagnostic patterns and growing awareness among clinicians regarding the early onset of OAB components in BPH, prompting a proactive multidimensional approach.

The prescription trends identified in this study show strong concordance with international and domestic guidelines. These guidelines recommend 5‐ARI/AB combinations for patients with enlarged prostates and cautiously support the use of anticholinergic or β3‐agonist add‐ons for patients predominantly experiencing storage symptoms. Notably, the 2023 AUA guidelines now emphasize the preferential use of β3‐agonists over anticholinergics, citing better tolerability in older adults; these recommendations mirror our observation of increasing mirabegron use and declining anticholinergic prescriptions. This evolving trend suggests that Korean national guidelines should be updated to better reflect the age‐sensitive risk–benefit balance of OAB pharmacotherapy.

This study had some limitations. First, potential inaccuracies in diagnostic coding may have affected the identification of BPH cases. Second, the study lacked detailed clinical information, such as blood pressure, which can influence the selection of BPH pharmacotherapy; lifestyle factors (e.g., smoking, alcohol consumption, or physical activity) were similarly absent from the dataset, which may have influenced the analysis of risk factors. Third, policy changes or system‐level shifts that occurred during the study period, such as the introduction of 5‐ARI reimbursement criteria, may not have been fully reflected in the observed trends. Fourth, the analysis was based on the first prescribed drugs within 60 days of diagnosis and may not reflect treatment changes during that period due to symptom progression, adverse effects, or patient adherence. Finally, because this study used claims data, medications not reimbursed by the National Health Insurance, such as tadalafil and vibegron for BPH/LUTS, were not captured and thus excluded from the analysis despite their growing clinical importance.

Nonetheless, the use of national claims data provided valuable insights into the real‐world prescription patterns of BPH treatment. Stratification by age and year allowed for a detailed understanding of evolving treatment trends, including the adoption of newer medications and the shift toward OAB combination therapy, aligning with updated clinical guidelines. Future research should focus on assessing the long‐term safety, efficacy, compliance, and cost‐effectiveness of BPH medications, including tadalafil, vibegron, and fixed‐combination drugs that are not yet covered by insurance in South Korea. Overall, this retrospective analysis of treatment‐naïve patients in South Korea revealed that AB monotherapy was the most common initial treatment for BPH between 2015 and 2020. However, the increasing use of 5‐ARI/AB and AB/OAB combination regimens, particularly among older age groups, suggests a clinical shift toward more personalized and effective therapeutic strategies. These findings provide real‐world insights into prescribing behaviors and underscore the need for an ongoing evaluation of treatment effectiveness and policy considerations in BPH management.

## Author Contributions

Y.H.K. and N.K.J. conceived and designed the study. Y.H.K. and N.K.J. performed the analysis. Y.H.K. drafted the manuscript. All authors participated in writing the manuscript and approved the final version to be submitted for publication.

## Ethics Statement

This study was performed using a HIRA dataset that did not contain any personal patient information and was exempt from review by the Institutional Review Board of Pusan National University College in Korea (PNU IRB/2024_149_HR).

## Conflicts of Interest

The authors declare no conflicts of interest.

## Supporting information


**Table S1:** Comorbidity definitions based on KCD‐6 to KCD‐8 codes.
**Table S2:** List of medication codes.
**Figure S1:** Prescription trends of initial treatment use by drug regimen.

## Data Availability

The data used in this study were provided by HIRA with permission. Requests to access the data should be directed to HIRA (https://opendata.hira.or.kr).
