# Supplementary material for: Prescription Trends of Initial Pharmacotherapy for Benign Prostatic Hyperplasia Among Treatment‐Naïve Patients in South Korea: A Retrospective Analysis
Source: Low Urin Tract Symptoms. 2025 Sep 5;17(5):e70030. doi: 10.1111/luts.70030 (PMC12411890; doi:10.1111/luts.70030)
Supplement: Supplementary file 1 — Table S1: Comorbidity definitions based on KCD‐6 to KCD‐8 codes. Table S2: List of medication codes. Figure S1: Prescription trends of initial treatment use by drug regimen. [file LUTS-17-e70030-s001.docx]

Table S1. Comorbidity definitions based on KCD-6 to KCD-8 codes

| Comorbidities | KCD-6 to KCD-8 codes |
| --- | --- |
| Hypertension | I10, I11, I12, I13, I15 |
| Atrial fibrillation | I48 |
| Heart failure | I50 |
| Ischemic heart disease | I21, I22, I23, I24, I25 |
| Diabetes mellitus | E11, E12, E13, E14 |
| Peripheral vascular disease | I70, I73 |
| Dyslipidemia | E78 |
| Transient ischemic attack | G45 |
| Renal failure | N03, N04, N05, N17, N18, N19, Z49, Z94.0, Z99.2 |
| Angina pectoris | I20 |
| Cerebrovascular disease | I63, I64 |
| Gout/hyperuricemia | M10, E79.0 |
| Osteoporosis | M80, M81, M82 |

KCD, Korean Classification of Diseases

Table S2. List of medication codes

| Therapeutic Class | Drug | Korean Drug Codes |
| --- | --- | --- |
| α1-Adrenergic receptor blockers | Terazosin | 235501ATB, 235502ATB, 235503ATB |
|  | Doxazosin | 149101ATB, 149102ATB, 149104ATR, |
|  | Tamsulosin | 234601ACR, 234601ATD, 234601ATR, 234602ACR, 234603ACR, 234603ATD, 234603ATR |
|  | Alfuzosin | 104803ATR |
|  | Silodosin | 504202ACH, 504202ATB, 504203ACH, 504203ATD, |
|  | Naftopidil | 614201ATB, 614202ATB, 614203ATB |
| 5α-Reductase Inhibitors | Finasteride | 159001ATB |
|  | Dutasteride | 458801ACS, 458801ATB |
| Anticholinergics | Propiverine | 219701ATB, 219702ATB |
|  | Oxybutynin | 207001ATB, 207001ATR, 207005ATR, |
|  | Flavoxate | 159201ATB |
|  | Fesoterodine | 503801ATR, 503802ATR |
|  | Tolterlodine | 357101ATB, 357102ACR, 357102ATB, 357103ACR, 357103ATR |
|  | Trospium | 245502ATB, 245501ATB, 245503ATB |
|  | Solifenacin | 493801ATB, 493801ATD, 493802ATB, 493802ATD, 655601ATB, 655602ATB, 657401ATB, 657402ATB |
|  | Imidafenacin | 505801ATB, 505802ATD |
| β3-Agonist | Mirabegron | 625701ATR, 625702ATR |


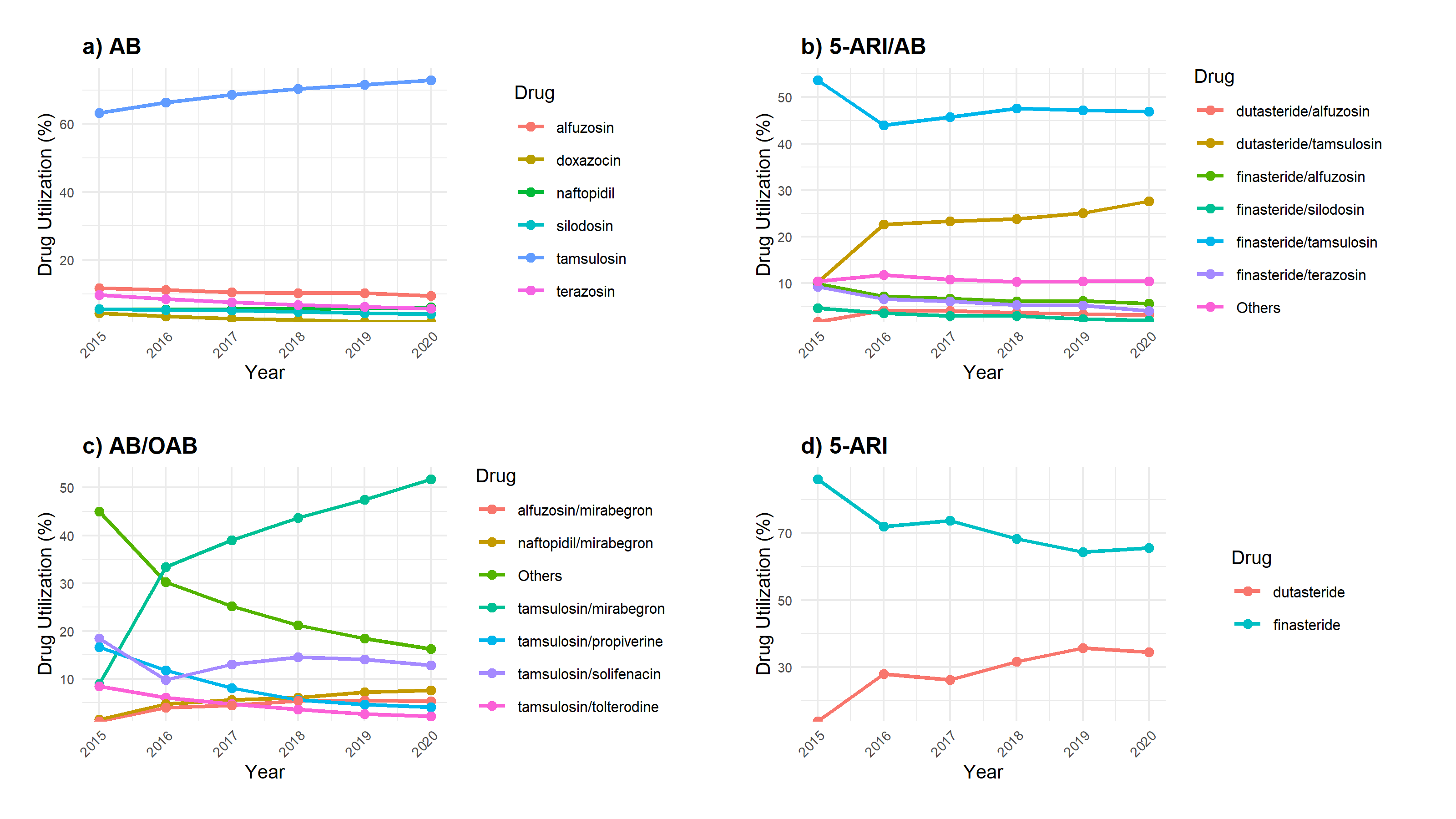


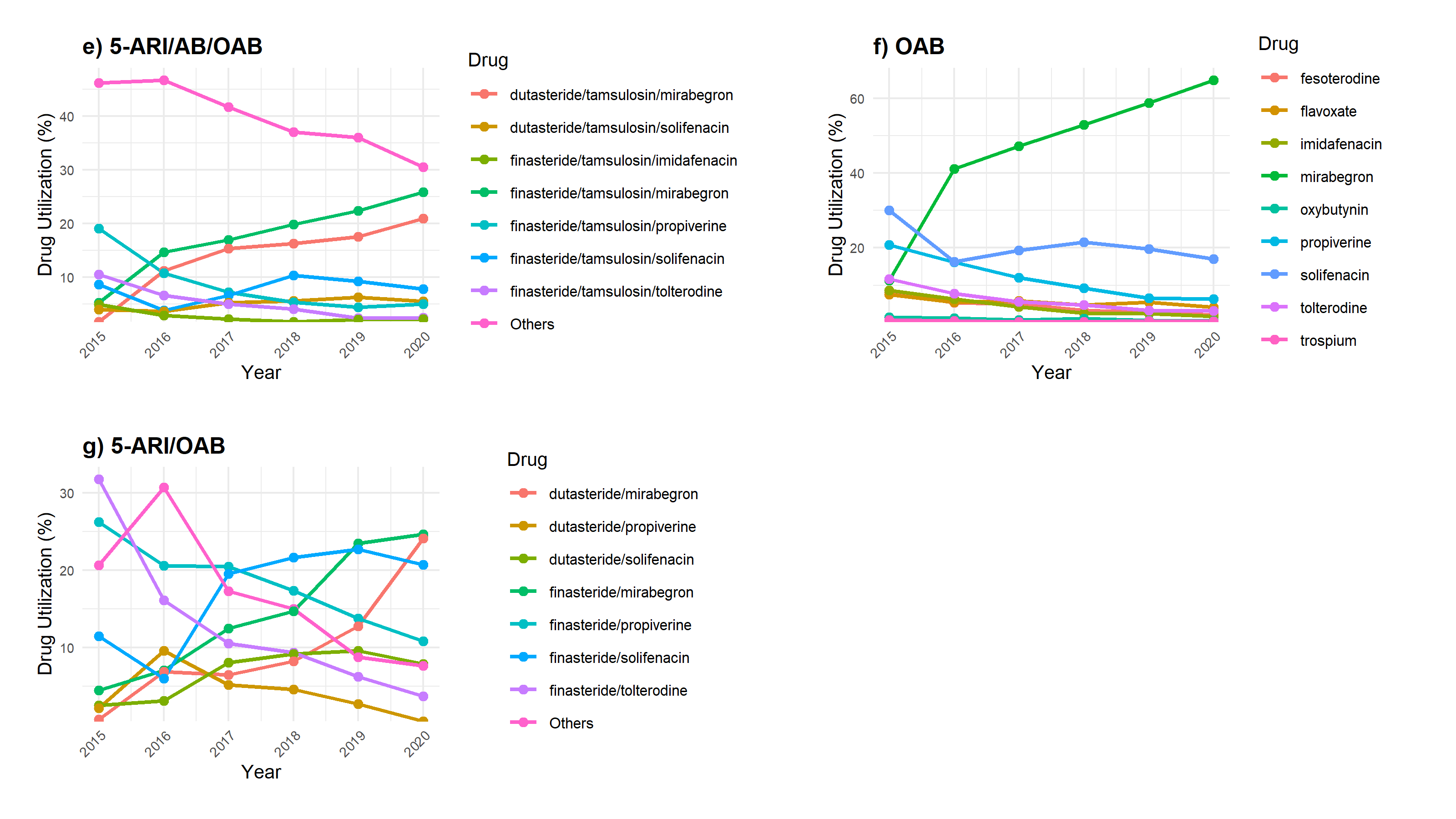


Figure S1. Prescription trends of initial treatment use by drug regimen

AB, α-blockers; 5-ARI, 5α-reductase inhibitors; OAB; overactive bladder agents
